# Shining a light on endometriosis: time to listen and take action

**DOI:** 10.1186/s12916-023-02820-y

**Published:** 2023-03-22

**Authors:** BMC Medicine

**Affiliations:** 4 Crinan Street, N19XW London, UK

Endometriosis, an often severe and common chronic inflammatory condition, affects around 1 in 10 women of reproductive age worldwide. It can have devastating effects on women’s physical and emotional well-being, quality of life, and reproductive health. Across the globe, March has been declared Endometriosis Awareness Month for a reason—it is time to increase awareness and education to allow for early diagnosis and eliminate the stigma surrounding this often-misunderstood condition. This editorial will shed light on endometriosis and explore why it is important to start listening and take action.

Endometriosis is characterized by tissue that normally lines the inside of the uterus growing outside, usually in the pelvic area. During the menstrual cycle, this displaced tissue continues to thicken, break down, and bleed, just like the tissue inside the uterus, causing a chronic inflammatory reaction that may result in scar tissue formation. Oftentimes, the endometrial tissue grows on pelvic organs such as the ovaries, fallopian tubes, and the outside of the uterus, resulting in fibrotic lesions and adhesions. Notably, the extent of endometrial lesions does not necessarily correlate with the severity of symptoms. Patients can experience severe symptoms despite the absence of visibly large lesions and vice versa, and they can even be asymptomatic.

Patients with endometriosis often suffer from pain in diverse manifestations, such as chronic pelvic pain, painful periods, and pain during intercourse, ranging from mild discomfort to severe pain. Some patients may experience heavy or irregular menstrual bleeding and fatigue, possibly resulting from chronic pain and inflammation often associated with this condition. Even worse, endometriosis can compromise reproductive health and cause infertility, leading to problems in getting pregnant.

These symptoms vary broadly, and not all endometriosis patients may experience them with the same intensity. Some patients may not experience any symptoms at all, putting them even more at risk for a delayed diagnosis and treatment. Symptoms may also change over time and often, but not always, improve after menopause.

The symptomatic ambiguity and the lack of awareness of endometriosis among both patients and healthcare providers often lead to underdiagnosis and prevent timely treatment of this condition, which has significant social, public health, and economic implications. Severe pain, fatigue, and infertility can all contribute to a significantly impaired quality of life for those affected by endometriosis. In the worst cases, the debilitating pain prevents them from pursuing regular day-to-day activities and attending school or work. Moreover, their sexual health and partnerships might be affected by painful intercourse and infertility.

Consequently, those affected often develop mental health problems and may suffer from depression or anxiety, accompanied by feelings of frustration, loneliness, and even shame, leading to a downward spiral and complex multimorbidities. Too often, patients get relief from their symptoms only after spending years with unsuccessful consultations or continue to suffer in silence as they interpret their symptoms as a normal part of their menstruation, which their healthcare providers may echo. For example, in the UK, more than half of people do not know about endometriosis, including 62% of women between the age of 16 and 24. But why is there such low awareness despite the high prevalence and devastating consequences?

The underlying problems are diverse and spread across different levels. At the scientific level, we must acknowledge that endometriosis remains an underresearched condition. Research efforts towards a better understanding of the pathophysiology and diagnostic treatment options are progressing only slowly. At the societal level, the situation is worsened by the normalization of women’s pain and persisting stigmatization around menstrual issues, which becomes even more problematic when entering the clinical level: Many people, including healthcare providers, may dismiss the symptoms of endometriosis as “normal” menstrual pain, leading to a delay in diagnosis and treatment. Additionally, the stigma surrounding menstruation and reproductive health can add barriers to seeking help, making those affected endure the consequences silently.

Therefore, we urgently need approaches towards better disease management, education, and awareness involving all relevant stakeholders to eliminate stigmatization and provide timely diagnosis and treatment. It is important to note that there is no cure at the moment. Still, good management options, including pain management, hormone therapy, or surgery, can significantly improve patients’ lives. For example, studies suggest a 62.5% improvement or resolution of pain 6 months after laparoscopy, a standard surgical treatment to remove endometrial tissue, with 90% still improved 1 year post-surgery. However, the average time to diagnosis is lengthy, and it can take over 7 years for patients to receive a proper diagnosis and, thus, treatment.

Simple solutions seldom resolve complex problems. Improving the lives of patients with endometriosis will require a multifaceted approach that involves the scientific community, healthcare providers, and society at large by focusing on the following:

- **Research funding**: We must invest in endometriosis research to build knowledge about the pathophysiology and develop new treatments. Advocacy groups and government agencies should be encouraged to fund studies to increase our understanding of the condition and provide better treatment options.
- **Medical education**: Medical schools and residency programs should include more information about endometriosis and its symptoms to ensure that healthcare providers can recognize the condition earlier and provide timely and appropriate care.
- **Patient advocacy and public awareness campaigns**: Support groups for people with endometriosis should be encouraged to share information and resources on various platforms, such as social media and blogs. Providing an open forum for exchange will help increase the chances of early diagnosis and alleviate patients’ mental burden by making them feel heard and connected.

Working collaboratively, patients, advocates, healthcare providers, and researchers can significantly improve care for those affected by endometriosis and break new ground. The Endometriosis Awareness Promotion Project (EAPP), a multinational and multicentre epidemiological study, is an excellent example of how collaborative efforts can lead to meaningful action: This study explores the key aspects of menstrual pain and endometriosis, including its personal and social impact. Promising first results suggest that an educational scheme can improve young women’s awareness and knowledge of endometriosis, highlighting the role of education for better outcomes.

Although initiatives such as the EAPP are encouraging, women’s health has been neglected for far too long. To shake things up and eventually improve the lives of those impacted by endometriosis, we must continue raising awareness at all levels. These efforts will pave the way for achieving the core goals proclaimed by the UK Endometriosis Association: reducing diagnosis time and providing access to treatment and support for patients. By taking action against endometriosis, we can make a huge difference in the lives of those affected and empower them by supporting their human right to the highest standard of sexual and reproductive health.

